# 1265. *In Vitro* Activity of Aztreonam-Avibactam against *Klebsiella pneumoniae* Isolates Analyzed by Epidemic Lineage and Hypervirulence Factors Collected in China as Part of the ATLAS Global Surveillance Study in 2019

**DOI:** 10.1093/ofid/ofab466.1457

**Published:** 2021-12-04

**Authors:** Mark Estabrook, Krystyna Kazmierczak, Francis Arhin, Daniel F Sahm

**Affiliations:** 1 IHMA, Schaumburg, Illinois; 2 IHMA, Inc., Schaumburg, Illinois; 3 Pfizer Canada, Kirkland, Quebec, Canada

## Abstract

**Background:**

Hypervirulent *Klebsiella pneumoniae* (hvKp), unlike classical *K. pneumoniae* (cKp), are often responsible for community-acquired infections in otherwise healthy individuals. The acquisition of hypervirulence genes by sequence type 11 (ST11) carbapenem-resistant (CR) Kp endemic in Asia is a grave threat. Aztreonam-avibactam (ATM-AVI) is a monobactam combined with a β-lactamase inhibitor for the treatment of infections caused by Enterobacterales isolates that carry Class A, B, C and some Class D β-lactamases.

**Methods:**

487 *K. pneumoniae* isolates were collected from 17 sites in China in 2019 as a part of the ATLAS global surveillance study. 220 isolates with MICs >1 µg/ml to meropenem (MEM), ceftazidime or ATM were selected for whole genome sequencing (Illumina Hiseq 2x150 bp reads). Analyses were carried out using the CLC Genomics Workbench (Qiagen). Presence of the aerobactin synthesis locus differentiated hvKp and cKp. Antimicrobial susceptibility was determined by CLSI broth microdilution.

**Results:**

Of the 487 isolates, MIC_90_ values for ATM-AVI (0.5 µg/ml; Table) were lower than those for any comparator tested, with only two isolates testing with MIC >4 µg/ml. Of the isolates sequenced, 82/220 (37.3%) were ST11. 53/82 (64.6%) of these ST11 isolates were hvKp (ATM-AVI, MIC_90_ 1 µg/ml; range, 0.25-4 µg/ml) and showed percentages of susceptibility < 90% to three last-line agents (0% MEM-susceptible (S); 18.9% amikacin (AMK)-S; 88.7% tigecycline (TGC)-S). Isolates of other STs (Non-ST11) were less frequently identified as hvKp (24/138, 17.4%) and more Non-ST-11 hvKp and cKp alike were S to MEM and AMK relative to isolates of ST11 (75.0-86.8% MEM-S; 83.3-96.5% AMK-S). Likewise, the ATM-AVI MIC_90_ value (0.25 µg/ml) was 4-fold lower for Non-ST11 isolates.

Results Table

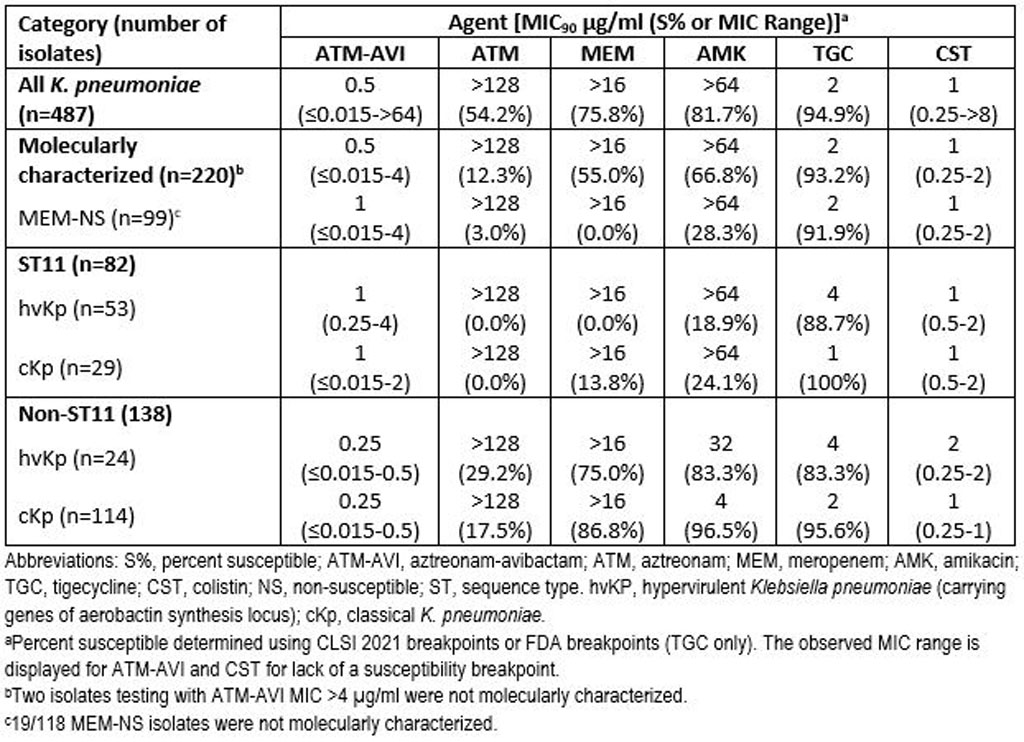

**Conclusion:**

CR ST11 hvKp represented at least 10.9% of the collected Kp isolates. ATM-AVI retained potent *in vitro* activity against these isolates which displayed resistance to a range of last-line agents. CST and TGC also displayed some activity but are limited in utility due to nephrotoxicity and poor accumulation in blood, respectively. The spread of virulence factors leading to the complicated clinical presentation of hvKp infection into multidrug-resistant lineages warrants continued surveillance.

**Disclosures:**

**Mark Estabrook, PhD**, **IHMA** (Employee)**Pfizer, Inc.** (Independent Contractor) **Krystyna Kazmierczak, PhD**, **IHMA** (Employee)**Pfizer, Inc.** (Independent Contractor) **Francis Arhin, PhD**, **Pfizer, Inc.** (Employee) **Daniel F. Sahm, PhD**, **IHMA** (Employee)**Pfizer, Inc.** (Independent Contractor)

